# Intersegmental Coordination in Patients With Total Knee Arthroplasty During Walking

**DOI:** 10.3389/fbioe.2022.839909

**Published:** 2022-02-24

**Authors:** Yingpeng Wang, Shuyan Qie, Yingqi Li, Songhua Yan, Jizhou Zeng, Kuan Zhang

**Affiliations:** ^1^ Department of Rehabilitation, Beijing Rehabilitation Hospital, Capital Medical University, Beijing, China; ^2^ School of Biomedical Engineering, Capital Medical University, Beijing, China; ^3^ Beijing Key Laboratory of Fundamental Research on Biomechanics in Clinical Application, Capital Medical University, Beijing, China; ^4^ Department of Orthopedics, Beijing Luhe Hospital, Capital Medical University, Beijing, China

**Keywords:** total knee arthroplasty, intersegmental coordination, continuous relative phase, statistical parametric mapping, gait analysis

## Abstract

Precise identification of deficient intersegmental coordination patterns and functional limitations is conducive to the evaluation of surgical outcomes after total knee arthroplasty (TKA) and the design of optimal personalized rehabilitation protocols. However, it is still not clear how and when intersegmental coordination patterns change during walking, and what functional limitations are in patients with TKA. This study was designed to investigate lower limb intersegmental coordination patterns in patients with knee osteoarthritis before and after TKA and identify how intersegmental coordination of patients is altered during walking before and after TKA. It was hypothesized that 6-month after TKA, intersegmental coordination patterns of patients are improved compared with that before TKA, but still do not recover to the level of healthy subjects. Gait analysis was performed on 36 patients before and 6-month after TKA and on 34 healthy subjects. Continuous relative phase (CRP) derived from the angle-velocity phase portrait was used to measure the coordination between interacting segments throughout the gait cycle. Thigh-shank CRP and shank-foot CRP were calculated for each subject. Statistical parametric mapping (SPM), a one-dimensional analysis of the entire gait cycle curve, was performed directly to determine which periods of the gait cycle were different in patients and healthy subjects. Six-month after TKA, thigh-shank CRP was significantly higher during 5–12% of the gait cycle (*p* = 0.041) and lower during 44–95% of the gait cycle (*p* < 0.001) compared with healthy subjects, and was significantly higher during 62–91% of the gait cycle (*p* = 0.002) compared with pre-operation. Shank-foot CRP was significantly lower during 0–28% of the gait cycle (*p* < 0.001) and higher during 58–94% of the gait cycle (*p* < 0.001) compared with healthy subjects, and was significantly lower during 3–18% of the gait cycle (*p* = 0.005) compared with pre-operation. This study found that patients exhibited altered intersegmental coordination during the loading response and swing phase both before and after TKA. Six-month after TKA, the thigh-shank coordination was partially improved compared with pre-operation, but still did not recover to the level of healthy subjects, while there was no improvement in the shank-foot coordination pattern after TKA compared with pre-operation. CRP combined with SPM methods can provide insights into the evaluation of surgical outcomes and the design of rehabilitation strategy.

## Introduction

Total knee arthroplasty (TKA) is an effective procedure for the treatment of advanced knee joint diseases (Yoshida et al., 2012; Levinger et al., 2013; Bonnefoy-Mazure et al., 2017). Postoperative physical function assessment is essential for the evaluation of therapeutic effects and the formulation of rehabilitation strategies. Gait analysis is an objective evaluation method that has been widely used in the evaluation of motor function in patients with knee osteoarthritis (OA) and TKA (Milner, 2009; Yoshida et al., 2012; Levinger et al., 2013; Alice et al., 2015; Bonnefoy-Mazure et al., 2017; Bączkowicz et al., 2018). However, current gait evaluation mainly focuses on isolated individual joint kinematics and kinematics at specific events or on summary metrics, such as maximum and minimum, which may be insufficient for fully describing the coordination involved in the completion of a movement task (Chiu and Chou, 2013; Chiu et al., 2013). Walking is a complex task that imposes a high demand on the motor control system to generate coordinated limb movements to achieve a smooth and accurate gait pattern (Ogaya et al., 2016; Salehi et al., 2020). The deficit in one joint can affect the coordination of the entire lower limb chain (Milner, 2009). Lower limb intersegmental coordination can be a good indicator of motor control mechanisms during walking (Ogaya et al., 2016; Salehi et al., 2020).

Continuous relative phase (CRP) derived from the angle-velocity phase portrait and containing both spatial and temporal information related to two segments or joints has been used to measure the coordination between interacting segments or joints throughout the gait cycle (Chiu et al., 2010; Ogaya et al., 2016). However, few studies have examined intersegmental coordination of patients with TKA during walking, and it remains unclear whether and how TKA causes the changes of intersegmental coordination in patients. A better understanding of the alterations of coordination patterns after TKA could aid clinicians to evaluate therapeutic effects and design rehabilitation strategies.

In addition, a common practice in biomechanical analysis is focusing on specific events or summary metrics, rather than considering the entire measurement domain, which may miss differences during other instances of the task or along the time dimension, resulting in increased Type I or Type II error (Pataky et al., 2013; Donnelly et al., 2017). Statistical parametric mapping (SPM) is a multidimensional data analysis technique that allows the presentation of statistical outputs in the original time series, providing an understanding of temporal regions where significant differences may occur (Pataky, 2012). It has proven to be applicable to a variety of biomechanical datasets including joint angle waveforms (Pataky, 2012; Pataky et al., 2013; Pataky et al., 2016; Donnelly et al., 2017; Pincheira et al., 2019; Papi et al., 2020; Moisan et al., 2021). Analyzing the entire gait waveform directly can enhance the understanding of the strategies adopted for the full gait cycle (Donnelly et al., 2017). Precise identification of deficient gait phases is conducive to the design of optimal personalized rehabilitation protocols.

It is still not clear how and when intersegmental coordination patterns change during walking, and what functional limitations are after TKA. Therefore, the aim of this study was to combine the CRP and SPM methods to investigate lower limb intersegmental coordination patterns, and to identify how intersegmental coordination of patients is altered during walking before and after TKA. It was hypothesized that 6-month after TKA, intersegmental coordination patterns of patients are improved compared with that before TKA, but still do not recover to the level of healthy subjects.

## Methods

### Subjects

Thirty-six patients (12 males and 24 females; age 64.9 ± 5.6 years; weight 70.1 ± 10.0 kg; height 161.3 ± 7.5 cm) planning to undergo unilateral TKA due to severe knee OA and 34 healthy subjects (15 males and 19 females; age 60.9 ± 6.3 years; weight 65.9 ± 10.6 kg; height 166.9 ± 7.4 cm) with no known knee pathology were enrolled in this study. All patients presented with medial compartment knee OA and had knee pain in the affected limb with Kellgren & Lawrence disease severity grades of III or IV. The contralateral limb exhibited no or milder knee OA. Exclusion criteria for all subjects included a history of lower limb or back surgery and neurological or orthopedic disorders other than knee OA that could affect gait. Patients were also excluded if they were unable to walk a short distance without technical aids. All healthy subjects were examined for medical history and underwent clinical examination, excluding subjects with lower extremity injury, disease or surgical history, and other conditions that may affect their gait. Approval was obtained from the Ethics Committee of Capital Medical University. All participants provided written informed consent.

### Total Knee Arthroplasty

Total knee arthroplasty was performed by the same senior chief physician using a posterior stabilized fixed platform prosthesis with the patella preserved. A median approach in front of the knee was taken and the articular cavity was entered through the medial side of the patella. Anterior and posterior cruciate ligaments were excised, femoral intramedullary positioning was performed, and tibial extramedullary positioning was performed. Osteotomy was performed according to the recommended standard of the prosthesis. The patient received standard rehabilitation training in the hospital after surgery and was instructed to conduct self-rehabilitation training at home after discharge. Patients were followed up for 6 months after surgery.

### Clinical Evaluation

Clinical evaluation was performed using the Hospital for Special Surgery Knee Score (HSS), the American Knee Society Score (KSS), the WOMAC score, and the knee range of motion (ROM) measures before and 6 months after TKA. HSS is a commonly used score for patients with TKA, that is scored between 0 and 100, where 0 indicates extreme symptoms and 100 indicates no symptoms (Insall et al., 1976). KSS is a joint-specific instrument consisting of knee and function scores with 100 points for each subscale, where a higher score indicates better health (Insall et al., 1989). WOMAC consists of 24 disease-specific questions with a total score of 240, and higher scores indicate more problems (Bellamy et al., 1988).

### Gait Analysis

A six-camera three-dimensional motion capture system (Motion Analysis Inc., Santa Rosa, CA, United States) sampled at 120 Hz was used to collected gait data. The modified Helen Hayes marker set with 19 markers was used to define body segments. A static trial was captured to identify joint center locations and to calculate the segment coordinate systems. Subjects were asked to walk a few trials along the test walkway at a comfortable speed to familiarize themselves with the experimental surroundings. In the formal test, a minimum of six walking trials was performed. All gait tests were performed by the same experienced professional. The raw data were filtered using a 6 Hz low-pass Butterworth filter. Gait data from the middle 3-m was selected for analysis to exclude the effects of acceleration and deceleration. OrthoTrak software (Motion Analysis Inc., Santa Rosa, CA, United States) was used to calculate gait spatiotemporal and kinematic parameters (Pincheira et al., 2019). Thigh, shank, and foot angles in the sagittal plane were calculated with respect to the vertical axis in each frame (Wang et al., 2021). Each gait cycle was time normalized to 100 data points.

### Continuous Relative Phase

A Matlab program (Matlab R2017b, the Mathworks Inc., Natick, MA, United States) was developed to calculate CRP using the Hilbert Transform approach as previously described (Lamb and Stöckl, 2014). The range of the segmental angles’ amplitude was first centered around zero, and then the phase angles were calculated based on the zero-centered segmental angle and its Hilbert transformation. The shank-foot CRP and thigh-shank CRP were calculated by subtracting the phase angles of the distal segment from those of the proximal segment. The average CRP curve calculated as the average for each time point across all gait cycles from each subject was used for subsequent statistical analysis. A more detailed description of the computational method is provided in a prior publication (Wang et al., 2021).

### Statistical Analysis

The sample size was calculated with data from our previous study (Wang et al., 2021) with intersegmental coordination in patients with knee OA during walking. The mean shank-foot average CRP during the early stance with the highest standard deviation and the smallest difference between groups was used (Cabral et al., 2018). The sample size was determined based on predicted power to detect a difference of 6.89° (SD 6.82°) between the groups with an expected effect size of 1.01, an alpha of 0.05 and 90% power. Based on calculations performed in G*Power (Faul et al., 2007), a minimum sample size of 22 subjects for each group was needed. Paired t-tests were performed to compare the clinical evaluation results before and 6 months after TKA. One-dimensional analysis of the entire gait cycle curve using SPM was performed directly to determine which periods of the gait cycle were different in patients and healthy subjects. Data from the left and right limbs of healthy subjects were averaged for subsequent comparison. A two-tailed independent sample SPM{t} test was used to determine the difference in the CRP curves between healthy subjects and both limbs of patients before and 6 months after TKA. A two-way Repeated Measure ANOVA was used to analyze the influence of surgery (pre-operation and post-operation) and limbs (affected/operated limb and unaffected/non-operated limb) on CRP curves in patients with TKA. If the interaction effect of surgery and limbs was statistically significant, post hoc comparisons were undertaken using the paired SPM{t} test with Bonferroni correction (alpha = 0.05/4). SPM analyses were implemented in Matlab software using open-access SPM1D scripts (http://spm1d.org/; Pataky, 2012).

## Results

### Clinical Evaluation

Six-month after TKA, all the HSS scores, KSS knee score, KSS function score, WOMAC score and knee ROM were significantly improved compared with pre-operation (Table 1).

**TABLE 1:** Clinical evaluation results before and 6-month after TKA.

—	Pre-operation	Six-month after TKA	t	p
HSS	51.72 ± 9.56	90.04 ± 5.77	−24.142	<0.001
KSS_knee	45.33 ± 7.09	95.67 ± 3.68	−37.319	<0.001
KSS_function	46.81 ± 14.74	80.28 ± 10.55	−10.173	<0.001
WOMAC	102.22 ± 18.05	27.07 ± 9.40	20.774	<0.001
ROM (°)	91.78 ± 19.83	115.32 ± 13.54	−6.490	<0.001

### Thigh-Shank Coordination Pattern

**FIGURE 1:**
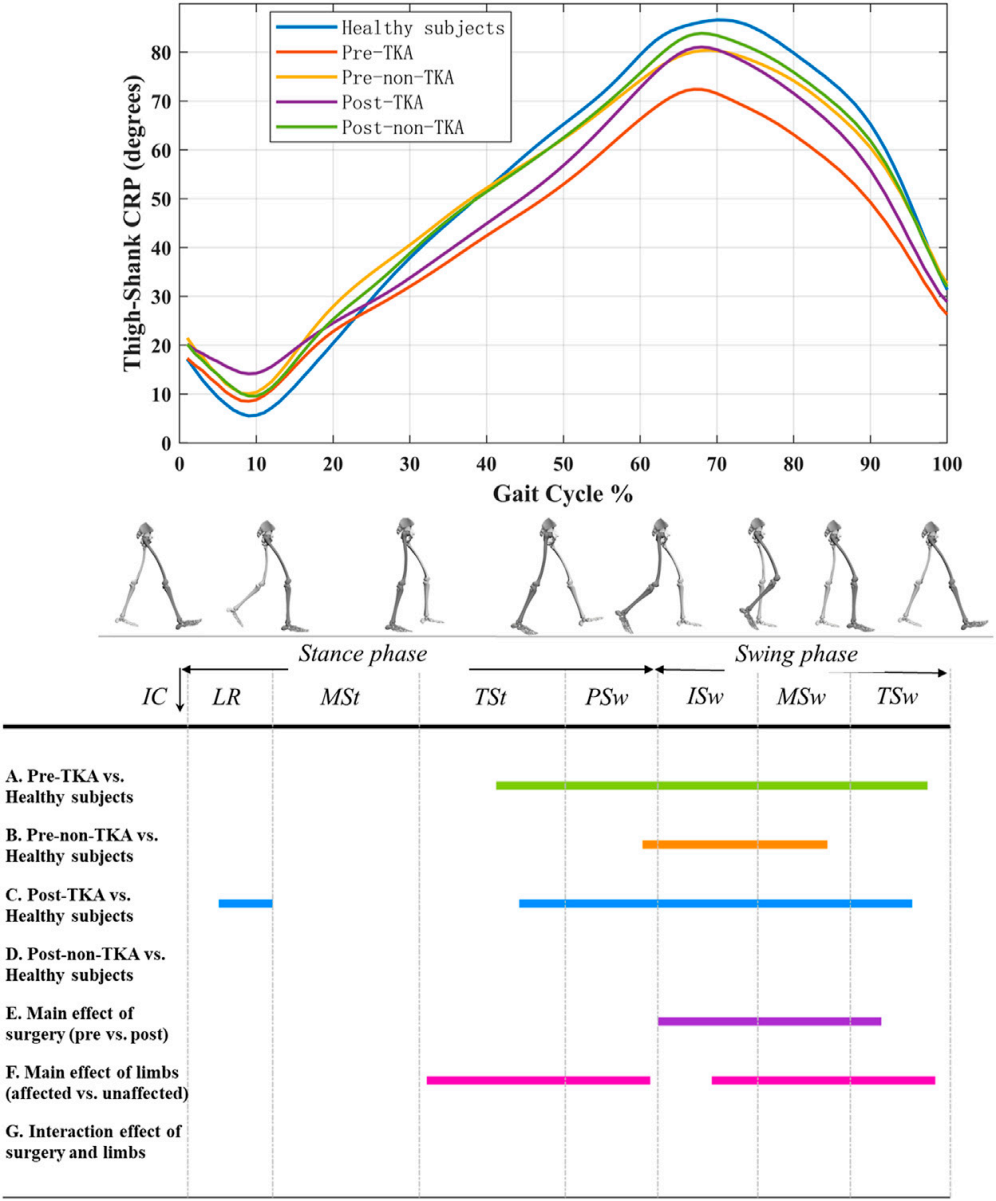
Thigh-shank CRP curves in patients before and after TKA and healthy subjects. A positive value indicates that thigh leads over shank, and a negative value indicates that shank leads over thigh. The colored lines in A ∼ G indicate that there are significant differences during this period when the two are compared.

Before TKA, thigh-shank CRP of the operated side was significantly lower compared to that of healthy subjects during 41–97% of the gait cycle (*p* < 0.001) (Figure 1A), and similar differences but shorter period was observed between non-operated side and healthy subjects (Figure 1B). Six-month after TKA, thigh-shank CRP of the operated side was significantly higher compared to that of healthy subjects during 5–12% of the gait cycle (*p* = 0.041) and significantly lower during 44–95% of the gait cycle (*p* < 0.001) (Figure 1C). There were no significant differences in thigh-shank CRP between the non-operated side of patients and healthy subjects throughout the gait cycle (Figure 1D). Compared with pre-operation, postoperative thigh-shank CRP was significantly increased during 62–91% of the gait cycle (*p* = 0.002) (Figure 1E). Thigh-shank CRP of the operated side was significantly lower during 32–61% and 69–98% of the gait cycle compared to that of non-operated side (*p* ≤ 0.003) (Figure 1F).

### Shank-Foot Coordination Pattern

**FIGURE 2:**
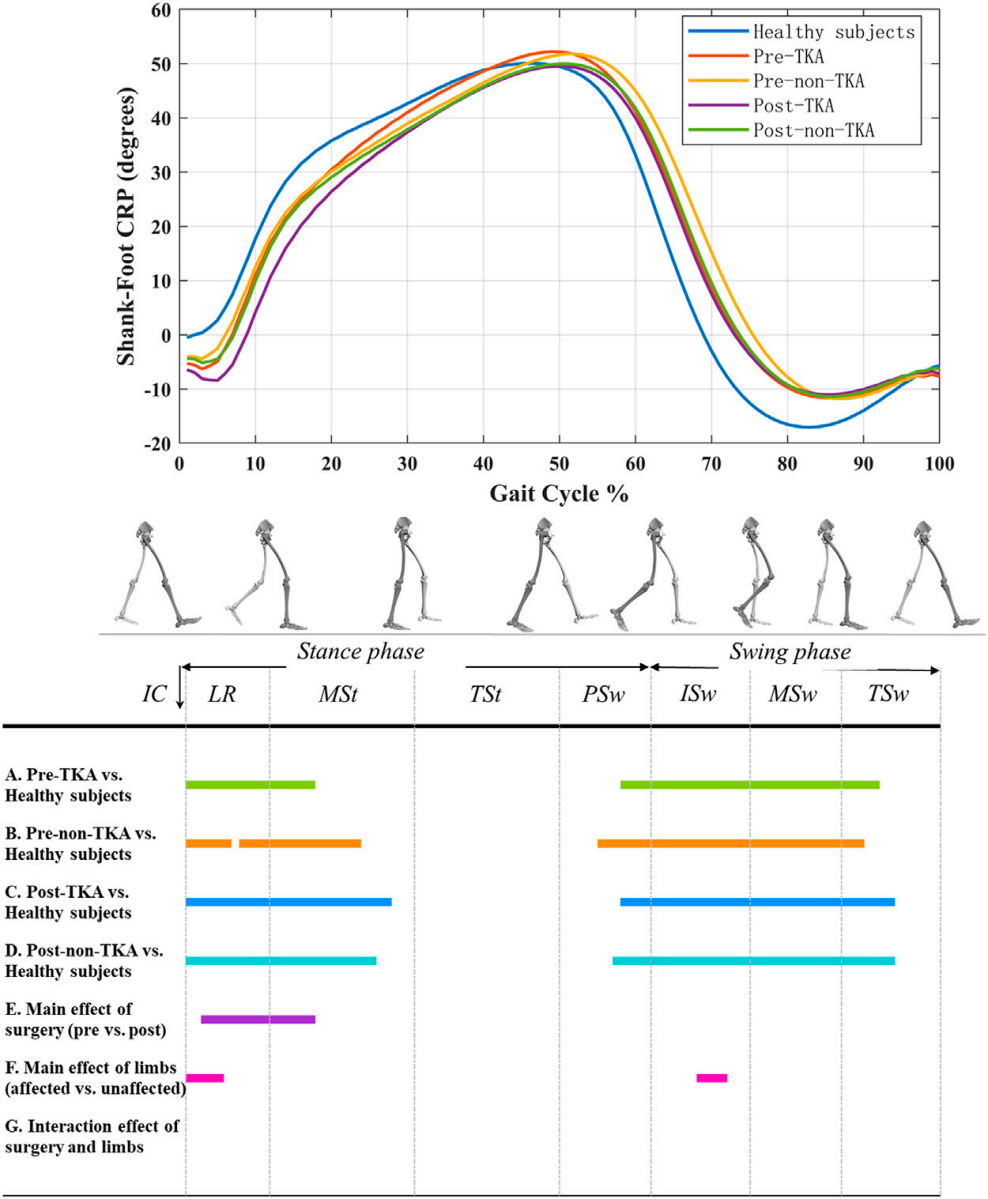
Shank-foot CRP curves in patients before and after TKA and healthy subjects. A positive value indicates that shank leads over foot, and a negative value indicates that foot leads over shank.

Before TKA, shank-foot CRP of the operated side was significantly lower compared to that of healthy subjects during 0–18% of the gait cycle (*p* < 0.001) and significantly higher during 58–92% of the gait cycle (*p* < 0.001) (Figure 2A). Similar differences were also observed between non-operated side and healthy subjects (Figure 2B). Six-month after TKA, shank-foot CRP of the both operated side and non-operated side were significantly lower compared to that of the healthy subjects during 0–26% of the gait cycle (*p* < 0.001) and significantly higher during 58–94% of the gait cycle (*p* < 0.001) (Figure 2C,D). Compared with pre-operation, postoperative shank-foot CRP of the operated side was significantly lower during 3–18% of the gait cycle (*p* = 0.005) (Figure 2E). Shank-foot CRP of the operated side was significantly lower during 0–6% and 68–72% of the gait cycle compared to that of non-operated side (*p* ≤ 0.044) (Figure 2F).

## Discussion

### Thigh-Shank Coordination Pattern

The results of this study showed that 6-month after TKA, the thigh-shank coordination pattern was significantly improved during 62–91% of the gait cycle compared to before TKA, but the lead of thigh over shank of the operated side in patients was still insufficient in the swing phase compared to healthy subjects. These results indicate that the thigh-shank coordination pattern of the operated side following TKA exhibited more in-phase coupling, and had not yet returned to the level of the healthy population. Previous studies have shown that the range of motion and the maximum flexion angle of the knee joint following TKA are significantly lower than that of healthy subjects, and patients still move with a knee-stiffening gait strategy (Alice et al., 2015; Rahman et al., 2015; Bonnefoy-Mazure et al., 2017) which can result in excessive in-phase coupling in the movement of thigh and shank. However, during normal walking more anti-phase is needed during the early swing to increase foot clearance and prepare for subsequently advancing the lower limbs. This thigh-shank coordination pattern in patients may also be related to the inadequate push off and gait velocity. Overall, patients still had abnormal knee kinematics and thigh-shank coordination patterns, even though knee pain and limited range of motion no longer restricted knee movement after TKA.

This study also found that thigh-shank CRP of the operated side following TKA was significantly increased compared to that of healthy subjects during 5–12% of the gait cycle, but there was no difference between preoperative patients and healthy subjects, which means that the operated side following TKA had an excessive lead of thigh over shank during the loading response. During this phase, a knee flexion excursion is required to absorb shock and power in order to decelerate the center of mass of the body (i.e., brake), prevent joint collapse, maintain forward propulsion, and provide vertical support (Worthen-Chaudhari et al., 2014). This can result in more in-phase coupling of thigh and shank movements. Numerous studies have shown that patients have a decreased knee flexion excursion during the loading response after TKA (Milner, 2009; Alice et al., 2015; Rahman et al., 2015; Bonnefoy-Mazure et al., 2017). The most plausible reason is that postoperative patients increase muscular co-contraction to maintain knee stability or adapt to the feeling of knee instability caused by cruciate ligament resection, proprioceptive defects, alterations in articular anatomical structure, and abnormal biomechanics of the prosthesis (Ro et al., 2018). Du et al. (Ro et al., 2018) believe that the cerebellum, which processes gait, recognizes the anomalous biomechanics of the knee joint induced by the implant, such as paradoxical anterior translation prior to rollback, as an altered gait pattern and suppresses knee movement via muscular co-contraction. Muscular co-contraction can limit the rotation of the shank and reduce the flexion angle of the knee joint, resulting in more anti-phase coupling of the thigh and shank movements. The maximum voluntary isometric contraction intensity of the muscles around the knee joint after TKA was significantly decreased compared to that of healthy subjects (Bade et al., 2010), indicating that the muscle strength of patients was weakened. This weakening may be caused by factors such as muscle disuse atrophy and surgical trauma. As a result, patients can only maintain the stability of the knee joint by tonic contraction of muscle groups, thus affecting the postoperative thigh-shank coordination pattern. This pattern of movement in patients may also result in more hip extension and ankle plantar during the loading response, leading to a reduction of shank-foot CRP. It is therefore necessary to use targeted rehabilitation training, such as muscle strength training and proprioception training, to adjust the gait coordination pattern of patients.

The non-operated side in patients exhibited significantly insufficient lead of thigh over shank during the swing phase before TKA. This insufficiency disappeared following TKA, indicating that the thigh-shank coordination pattern of the non-operated side had recovered to the level of the healthy subjects. This result further confirms the functional compensatory effect of the non-operated side to the knee dysfunction of the operated side. Previous studies have observed the compensation strategies by the unaffected limb in patients with unilateral knee OA (Lewek et al., 2006; Gustafson et al., 2019). The excessive compensation by the unaffected limb may increase its risk of articular cartilage damage and disease progression over time and should be taken as a warning (Gustafson et al., 2019). This could help to explain why numerous individuals with unilateral knee OA eventually develop symptoms of knee OA in the unaffected limb (Lewek et al., 2006).

Previous studies have shown that the angle of knee flexion and the motion amplitude of thigh and shank during walking of the non-operated side following TKA are less than that of healthy subjects (Rahman et al., 2015). However, when analyzing the thigh-shank coordination pattern in the present study, we observed that the timing and sequence of thigh and shank movement of the non-operated side following TKA were similar to that observed in healthy people. This finding also illustrates the difference between research examining single joints or intersegment coordination. Thus, the analysis of intersegmental coordination can provide insight into the coupling relationship of multiple segments, which is necessary and meaningful for assessing the walking ability of patients.

### Shank-Foot Coordination Pattern

Compared with healthy subjects, the lead of shank over foot before TKA in patients was significantly insufficient during the loading response and mid-stance, which was not improved after TKA. Especially, the operated side exhibited less lead of shank over foot compared to pre-operation, to the non-operation side, and to healthy subjects. This pattern of coordination is also due to the limitation of the knee flexion and the shank rotation during the loading response caused by the greater and longer muscular co-contraction around the knee joint, reflecting the functional compensation and adaptation of the ankle joint to the knee joint.

We also found that patients exhibited excessive lead of shank over foot both before and after TKA during the swing phase compared with healthy subjects. In addition, no improvement was observed postoperatively compared to preoperatively. For healthy subjects, the lead of shank over foot begins to decrease after reaching the maximum at about 50% of the gait cycle and changes to the lead of foot over shank at about 70% of the gait cycle, until reaching the negative maximum at about 80% of the gait cycle. The reason for this pattern is that, in mid-stance, the foot is placed flat on the ground with little movement until the heel lifts off the ground, and the foot rotation accelerates and gradually exceeds shank rotation. Before TKA, the timing of the heel and toe off the ground in patients was later than that of healthy subjects, which slow down the foot rotation, increasing the ankle dorsiflexion during the terminal stance, and reducing the ankle plantarflexion during the push off. This can be confirmed by previous works that reported patients with knee OA had higher maximum dorsiflexion during the stance and lower maximum plantarflexion during the push off compared to controls (Levinger et al., 2013; Ismailidis et al., 2021). A high-intensity concentric contraction caused by a rapid ankle plantarflexion generates sufficient power at the ankle joint during the propulsion and provides sufficient forward momentum for the leg swing (Winter 1989). The reduced ankle plantarflexion limits the ankle power generation during the push off, and therefore may limit forward progression of the body and diminish the momentum of the swing limb, which is also responsible for gait changes such as reduced step length and walking speed (Levinger et al., 2013). Studies have shown that patients with knee OA have reduced ankle power generation, even after adjusting for gait velocity (Levinger et al., 2013). After TKA, patients performed the same shank-foot coordination pattern during the swing phase as before the operation. The probable reason for this is the retention of the pre-operation gait pattern, as has been reported in previous studies (Smith et al., 2004; Milner, 2009; Levinger et al., 2013). The longitudinal pre- and post-surgery data presented by Smith et al. (Smith et al., 2004) indicated that pre-surgery gait patterns were still retained 18 months after surgery. Therefore, just because individuals are no longer suffering from pain at the knee and have the ability to move through a sufficient range of motion, it does not necessarily mean that they will spontaneously modify their gait to a more normal pattern. Consequently, therapeutic strategies should focus on not only rehabilitation designed to improve knee function but also gait retraining to optimize recovery following TKA.

### Clinical Significance

CRP can be used to measure the interactions between segments or joints throughout the gait cycle, enabling the assessment of the overall coordination profile during walking. CRP has been demonstrated to be capable of identifying the essential timing and sequencing of neuromuscular control over biomechanical degree of freedom for a smooth and efficient movement and is considered to be able to complement traditional gait analysis to provide insights into the complexity of human motor behavior (Chiu et al., 2010; Chiu and Chou, 2012; Chiu et al., 2013; Ogaya et al., 2016; Wang et al., 2021). SPM is a useful analysis method for clinicians because it is more intuitive than considering discrete variables alone and allows one to explore gait waveform data without a priori assumptions about when in the gait cycle statistical differences might occur, and can further provide objective evidence for best practice clinical decisions making (Pincheira et al., 2019). In this study, the combination of CRP and SPM methods was used to detect the differences in lower limb intersegmental coordination patterns between patients with unilateral TKA and healthy subjects throughout the gait cycle. The results highlight the deficiencies of coordination patterns in patients with TKA and in which phases of the gait cycle they occur. Individualized treatment planning could use these findings as a guideline and studies focusing on the effects of treatment could further help transfer this knowledge to everyday practice.

Abnormal thigh-shank and shank-foot coordination patterns were observed during the loading response. This may be related to the abnormal knee muscle control caused by knee instability, proprioceptive defects, weakened muscle strength, abnormal biomechanics of the prosthesis. Accordingly, clinicians should focus on the knee control training after TKA, especially the eccentric contraction of the quadriceps femoris to control the knee joint better during the loading response. Meanwhile, proprioceptive training should also be carried out as soon as possible due to the reduction of the patient’s perioperative ground activities and the lack of cruciate ligaments. Moreover, after TKA, shank-foot coordination pattern during the swing phase showed no improvement compared to pre-operation, which may be the retention of the preoperative gait pattern. Thus, gait training should be done as early as possible according to the condition of patients. Verbal cues and Rehabilitation equipment that provides real-time feedback via cameras and screens may be helpful for correcting the abnormal gait pattern of patients. In addition, given the substantial impairment and functional deficits present prior to knee surgery, as well as the additional trauma of surgery itself, more aggressive rehabilitation may be needed to remediate these impairments and functional deficits and restore gait coordination patterns closer to the levels of healthy adults. More interventional research following TKA is needed to address how this can be most effectively accomplished. The phases of the occurrence of coordination pattern defects in the gait cycle could assist in clarifying whether a targeted treatment or a more generalized one would be beneficial for a patient.

### Limitations

The interpretations of our study should be considered along with some limitations. Six months after TKA may not be sufficient for the recovery. Given a longer recovery time of more than 6 months, the intersegmental coordination pattern of patients may be better than that before surgery, and closer to that of healthy subjects. Some studies have shown that the recovery period after TKA is about 1 year, and that the gait of patients stabilized 1 year after surgery (Meier et al., 2008; Kiss et al., 2012). However, other studies have found that patients recovering from TKA typically plateau in strength and functional gains at 6-month, and it is believed that the degree of gait recovery within 6-month determines the final gait of patients, so functional rehabilitation training is recommended within 6-month (Bade et al., 2010). A longer follow-up is needed to confirm the results of this study. In this study, the rehabilitation process was not controlled, and patients were doing their own rehabilitation at home, which may lead to uneven rehabilitation effects and affect the recovery of knee function after the operation. A comfortable rather than controlled gait speed was selected in this study. Data on controlled gait speed may be less reflective of the subjects’ regular walking characteristics. In addition, it is unclear if kinematic alterations are a cause or a consequence of reduced gait velocity.

## Conclusion

The changes of intersegmental coordination pattern during the loading response and swing phase were identified in the surgical group before and after TKA compared to healthy subjects. Six-month after TKA, the thigh-shank coordination pattern was partially improved compared with that before TKA, but still did not recover to the level of healthy subjects, while there was no improvement in the shank-foot coordination pattern after TKA. Rehabilitative strategies may therefore need to focus not only on improving knee function, but also on gait coordination pattern retraining, to optimize recovery following TKA. The combination of CRP and SPM methods can be used to provide new insights into motor function assessment and the evaluation of surgical outcomes and the design of rehabilitation strategy.

## Abbreviations

Initial Contact; LR: Loading Response; MSt: Mid-Stance; TSt: Terminal Stance; PSw: Pre-Swing; ISw: Initial Swing; MSw: Mid-Swing; TSw: Terminal Swing.

## Data Availability

The raw data supporting the conclusions of this article will be made available by the authors, without undue reservation.
